# Targeting T Cell Subtypes for NAFLD and NAFLD-Related HCC Treatment: An Opinion

**DOI:** 10.3389/fmed.2021.789859

**Published:** 2021-11-18

**Authors:** Chunye Zhang, Ming Yang

**Affiliations:** ^1^Department of Veterinary Pathobiology, University of Missouri, Columbia, MO, United States; ^2^Department of Surgery, University of Missouri, Columbia, MO, United States

**Keywords:** T cell subpopulation, NAFLD, HCC, treatment, cytokines, chemokines

## Introduction

The increasing prevalence of non-alcoholic fatty liver disease (NAFLD), as well as its advanced stage non-alcoholic steatohepatitis (NASH) with the progression of liver inflammation and cell death with or without hepatic fibrosis, brings a heavy burden to public health (1). Non-alcoholic fatty liver disease is commonly associated with the incidence of obesity and diabetes (2, 3). In the United States, the prevalence of obesity raised from 30.5 to 42.4% from years 1999–2000 to years 2017–2018 as the Centers for Disease Control and Prevention (CDC) reported, and the prevalence of severe obesity also increased from 4.7 to 9.2% at this period. Recently, a new nomenclature of NAFLD, metabolic associated fatty liver disease (MAFLD), was recommended, which is thought to be more accurate to reflect the clinical pathogenesis of this disease with metabolic dysfunction (4, 5). There is no appropriate treatment for NAFLD up to date, except for early prevention via change of lifestyle (2, 6). Understanding the cellular and molecular pathogenesis of NAFLD and its relative advanced liver disease is helpful to define new potential targets for treatment.

Hepatic immunity plays a critical role in the pathogenesis of liver diseases (7, 8), including NAFLD, NASH, and end-stage of liver disease. Both hepatic innate and adaptive immune cells, as well as their interaction, orchestrate the progression of NAFLD and NASH (9). For example, the accumulation of activated hepatic B cells driven by gut microbiota impacted liver inflammation and fibrosis via modulating both intrahepatic innate and adaptive immunity during the progression of NASH (10). New functions of special types of T cells are reported to be associated with the progression of NAFLD and hepatocellular carcinoma (HCC) defined by the single-cell RNA sequencing (sRNA-seq) technology (11, 12). Here, we mainly focus on the latest investigation of the function of special types of T cells in NAFLD and NAFLD-related primary liver cancer.

## Factors Causing NAFLD and NAFLD-Related HCC Progression

Non-alcoholic fatty liver disease is an increasing factor that induces the development of HCC (13). The pathogenesis of NAFLD-related HCC progression remains to be clarified. The causing factors such as genetic factor (e.g., the genetic variant I148M of rs738409 in patatin-like phospholipase domain containing 3, PNPLA3) and epigenetic factors (e.g., histone deacetylase) for NAFLD and NASH may result in liver fibrosis and cirrhosis, and finally leading to the development of HCC (14–17). In addition, several other factors including environmental factors have been identified to be associated with NAFLD-related HCC progression (18), such as lipid metabolism (19), and dysregulation of gut microbiota (20). For example, dysregulation of lipid metabolism in NAFLD induced hepatic accumulation of linoleic acid and subsequent loss of CD4^+^ T cells due to an increase of reactive oxygen species (ROS) (21), resulting in an increased incidence of HCC. Clinical trial studies also showed that anti-programmed death-1 (PD-1) or anti-programmed death-ligand 1 (PD-L1) treatment decreased the overall survival (OS) of human patients with NASH-induced HCC compared to non-NASH-induced HCC patients (22). Cellular mechanism study demonstrated that stimulation with gut microbial extracts from NAFLD-related HCC subjects can increase the frequency of regulatory T cells (Tregs) and decrease the frequency of CD8^+^ T cells in human peripheral blood mononuclear cells (PBMCs), compared to treatment with bacterial extracts from non-NAFLD subjects (20), which indicates an important role of gut microbiota in modulating immunity in HCC microenvironment. In addition, peripheral PBMCs showed an immunosuppressive phenotype in human patients with NAFLD-related HCC compared to non-NAFLD and NAFLD-cirrhosis patients (20). Independent of these discussed causing factors for liver disease, T cells play an important role in the progression of NAFLD and NAFLD-related HCC. Thus, it is critically important to delicate the function of each subtype of T cells in NAFLD-HCC progression.

## Function of T Cells in NAFLD and NAFLD-Related HCC

### Function of CD8^+^ T Cells in NAFLD

LIGHT (tumor necrosis factor superfamily member 14, TNFSF14) expression in activated CD8^+^ T cells induced by feeding a choline-deficient high-fat diet (CD-HFD) promoted NASH and HCC progression in mice via interacting with lymphotoxin-β receptor (LTβR) in hepatocytes (23). CD8^+^ T cells were also increased in the livers of obese human patients with NASH and cirrhosis, which was positively correlated with hepatic stellate cell (HSC) activation, evidenced by the increased expression of α-smooth muscle actin (α-SMA) (24). In contrast, depletion of CD8^+^ T cells significantly reduced liver inflammation and HSC activation. A 3.5-fold increase of CD8^+^ T cells with high expression of cytotoxic interleukin (IL)-10 can also be found in obese mice while feeding a western diet (WD) compared to the chow diet (24). High expression of IL-10 may promote the progression of HCC (25). Another study also showed that impairing CD8^+^ T cell activation in mineralocorticoid receptor (MR)-deficient mice decreased liver steatosis in a methionine-choline deficient diet (MCD)-induced NASH model (26). Tumor development altered fatty acid partitioning in the fatty liver via inhibiting prolyl hydroxylase domain (PHD)3 expression, which results in function loss of cytotoxic CD8^+^ T cells and impaired anti-tumor function (27). Therefore, enhancing or reversing the role of CD8^+^ T cells in NAFLD may inhibit NAFLD-HCC progression. Here, we summarize some specific subpopulations of CD8^+^ T cells in NAFLD-related HCC.

### Function of CD8^+^ T Cells in NAFLD-Related HCC

#### PD1^+^CD8^+^ T Cells

Preclinical study showed that immunotherapy with anti-PD1 treatment increased the prevalence of exhausted PD1^+^CD8^+^ T cells with high mRNA expression of C-X-C motif chemokine receptor 6 (CXCR6) and tumor necrosis factor-alpha (TNF-α) in the liver of NASH mice, which was associated with impaired immune surveillance and increased incidence of NASH to HCC progression (22). Similar phenotypic and functional PD1^+^CD8^+^ T cells were found in livers from humans with NAFLD/NASH in this report. In addition, both anti-CD8 or anti-TNF plus anti-PD1 antibody treatments can ameliorate liver damage and inflammation and reduce HCC incidence compared to anti-PD1 treatment alone.

#### CXCR6^+^CD8^+^ T Cells

Liver-resident CXCR6^+^CD8^+^ T cells were increased in NASH mice fed a CD-HFD, and those CD8^+^ T cells expressed low activity of the Forkhead box protein O1 (FOXO1) transcription factor caused by high expression of IL-15 (28). In addition, the level of hepatic acetate was increased in NASH mice, which can cause auto-aggressive liver CXCR6^+^ CD8^+^ T cells to damage hepatocytes, resulting in liver injury. Furthermore, CXCR6^+^CD8^+^ T cells were also shown to increase in human NAFLD/NASH livers, as well as hepatic expression of CXCR6 (28).

#### Prf1^null^CD8^+^ T Cells

Perforin (Prf)-deficient mice on an MCD showed an increased accumulation and activation of CD8^+^ T cells expressing proinflammatory cytokines (e.g., interferon-gamma, or IFN-γ) compared to wild-type (WT) mice, but not CD4^+^ T cells (29). The increased IFN-γ levels are closely associated with liver dysfunction in human patients, including liver fibrosis, cirrhosis, and HCC (30). In contrast, an increase of cell proliferation antigen Ki67^+^CD8^+^ T cells producing IFN-γ in response to sorafenib treatment was associated with improved OS and progression-free survival (31).

### Function of CD4^+^ T Cells in NAFLD

Dysregulation of hepatic lipid metabolism in human NAFLD patients and mouse models induced a reduction of liver CD4^**+**^ T cells (21, 32). Fatty liver impairs the immunotherapeutic effects (33), such as RNA vaccine (e.g., M30-RNA vaccine) and antibody-mediated therapy [e.g., anti-OX40 (CD134) antibody]. Feeding a high-fat and high-calorie diet caused the proliferation of human CD4^+^ central and effector memory T cells in immunodeficient mice engrafted with human immune cells (HIL mice) compared to that in mice fed with a chow diet, which was associated with a significant increase of pro-inflammatory cytokines, such as IL-17A and IFN-γ (34). In addition, *in vivo* depletion of human CD4^+^ T cells in those mice can attenuate hepatic inflammation and fibrosis. In summary, these results show that CD4^+^ T cells play diverse roles in the development of NAFLD, liver fibrosis, and HCC. Thus, clarifying the function of each type of CD4^+^ T cell is necessary.

#### α4β7^+^ CD4^+^ T Cells

Recruitment of integrin α4β7^+^ CD4^**+**^ T cells to the liver was associated with NASH progression in F11r^−/−^ mice fed with WD, which was correlated with higher expression of its ligand mucosal addressin cell adhesion molecule 1 (MAdCAM-1) (35). Blocking integrin α4β7 prevented migration of CD4^**+**^ T cells, resulting in a significant decrease in liver inflammation and fibrosis. In addition, ablating β7 integrin or MAdCAM-1, as well as β7 integrin deficiency, can reduce concanavalin A (ConA)-induced hepatitis in mice, indicating the role of β7 integrin in liver injury (36).

#### Th17^+^ Cells

In the progression of NAFLD to NASH, hepatic IL-17^+^CD4^+^ T (Th17) cells were significantly increased, and the ratio of Th17 or Th2 to CD4^+^CD45RA^+^CD25^++^ resting Tregs (rTregs) was elevated in peripheral blood (37). Imbalance of hepatic Th17/Treg cells was also shown in NAFLD mice fed a HFD (38). The increased frequency of IL-17^+^ cells in total CD4^**+**^ T cells in NASH patients was positively correlated with a higher level of serum concentration of blood endotoxin (LPS) compared to either healthy subjects or non-alcohol fatty liver (NAFL) patients (39).

#### Treg Cells

The interaction of Foxp3^+^CD25^+^CD4^**+**^ T cells (Tregs) with other immune cells and hepatocytes plays a critical role in liver homeostasis and pathogenesis. Hepatocytes can engulf CD4^+^ T cells, preferable for Tregs, during liver inflammation to control T cell population, known as enclysis (40). The frequency of CD25^+^CD45^+^CD4^**+**^ T cells was increased in PBMCs of human NAFLD patients with advanced liver fibrosis, while the PD1^+^CD4^**+**^ T cells were decreased (41), which were significantly and negatively correlated with the ratio of serum fatty acid composition (44, 45).

Moreover, there are other subtypes of T cells that were found to be associated with the progression of NAFLD, such as Vδ2 T cells (42) and γδ T cells (43).

### Function of CD4^+^ T Cells in NAFLD-Related HCC

#### Treg Cells

Transcription factor Foxp3 can suppress glycolysis and induce oxidative phosphorylation to change metabolic profiles of Tregs to survive in low-glucose and high lactate environments (44). The proliferation of Tregs can suppress the function of cytotoxic CD8^**+**^ T cells against liver tumor cells, resulting in the progression of HCC both in mouse models and in human patients (45). A high ratio of effector CD4^+^ T cells/Treg showed a good prognostic for human HCC (31).

#### Th17 Cells

Th17 cells and the expression of IL-17a were positively associated with human fatty liver-associated HCC (46). *In vitro* study showed that macrophages are required to mediate IL-17 expression in naive CD4^+^ T cells through LPS/Toll-like receptor 4 (TLR4) signaling. Furthermore, intra-tumoral infiltration of Th17 cells promoted tumor growth via promoting angiogenesis and predicted a poor OS in HCC patients (47). In addition to inducing angiogenic factors (e.g., vascular endothelial growth factor/VEGF and prostaglandin E2/PGE2), Th17 cells can activate oncogenic IL-6/Stat3 signaling to enhance tumor growth (48).

### Function of Double-Negative T Cells in NAFLD-Related HCC

Double-negative T cells (DNT) defined by T-cell receptor (TCR)αβ^+^CD3^+^CD4^−^CD8^−^ T cells and consisting of 1–3% of peripheral T lymphocytes in mice and humans have been shown to play multiple roles in immune responses (49). Adoptive transfer of CD4^+^ T cells converted DNT was shown to reduce liver inflammation and fat accumulation inducing factors for NASH, by suppressing the infiltration of Th17 cells and M1 macrophages (8). Double-negative T cells can also inhibit the function of effector CD4^+^ T cells by impairing glucose metabolism and inhibiting mTOR signaling and the expression of inflammatory cytokines IL-17a and IFN-γ (50). Furthermore, DNT was shown to be higher in non-tumor-infiltrating lymphocytes compared to tumor-infiltrating lymphocytes in human HCC (51).

## Potential Treatment Options for NAFLD-Related HCC by Targeting on T Cells

**Figure 1:**
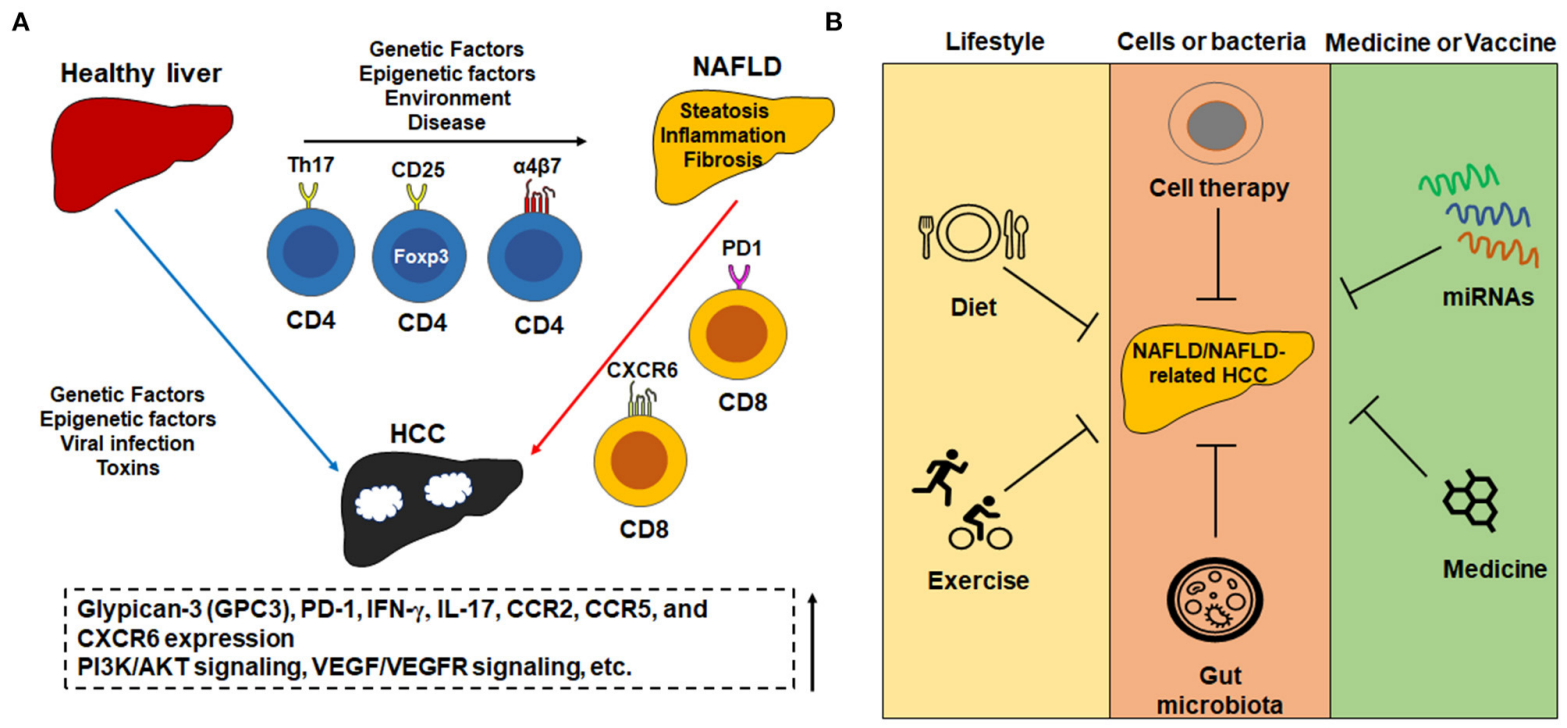
The important role of T cells in the pathogenesis of NAFLD and NAFLD-related HCC and potential treatment options. **(A)** Some important subtypes of T cells and molecules in the pathogenesis of NAFLD and NAFLD-related HCC. For example, Th17^+^CD4^+^, CD25^+^Foxp3^+^CD4^+^, and α4β7 integrin-positive CD4^+^ T cells increase in the progression of NAFLD, while PD1^+^CD8^+^ and CXCR6^+^CD8^+^ increase in NAFLD-HCC progression. The change of T cell population is associated with an increase of cytokines such as IL-17a and IFN-γ which accompany the progression of NAFLD. **(B)** The potential treatment options for NAFLD and NAFLD-related HCC include change of lifestyle, cells or bacteria-mediated therapy such as adoptive transfer of T cells, medicines or vaccines such as microRNA-mediated therapy.

Currently, there are some approved first- and second-line treatment options for HCC, which may be also applied in NAFLD-related HCC treatment. In 2008, sorafenib, a multi-kinase inhibitor against VEGF receptor (VEGFR), platelet-derived growth factor receptor (PDGFR), and RAF kinases (serine/threonine protein kinases), is the first approved systemic therapy by the U.S. FDA for patients with unresectable HCC (52). In 2018, lenvatinib, a multiple kinase inhibitor against the VEGFR1, VEGFR2, and VEGFR3 kinases, was approved by FDA for systemic treatment for unresectable advanced HCC (53). In 2020, PD-L1 inhibitor atezolizumab was approved by FDA in combination with bevacizumab (anti-VEGF monoclonal antibody) for adult patients with unresectable locally advanced or metastatic HCC without prior systemic therapy (54). In addition, there are some combined treatments such as nivolumab (anti-PD-1 monoclonal antibody) and ipilimumab (anti-cytotoxic T-lymphocyte-associated protein 4/CTLA4 monoclonal antibody) that may approve the outcomes (55). Here, we also review some treatment options by targeting T cells (Figure 1).

### T Cell-Mediated Treatment

A clinical trial shows that treatment with sorafenib, a protein kinase inhibitor, can increase Ki67^+^CD8^+^ T cells producing IFN-γ to improve progression-free survival and OS of human HCC patients (31). The VEGF/VEGFR signaling was involved in this effect, evidenced by improved sorafenib in combination with VEGFR antagonism (31).

A decrease of Tregs in visceral adipose tissue (VAT) is positively associated with NASH progression (56). Adoptively transfer (ACT) of Tregs from spleens of healthy mice to mice with diet-induced hepatic steatosis promoted liver steatosis with an increase of Tregs in VAT and a decrease of Th1 cells in various tissues (57). Adoptively transfer of Tregs did not impact other metabolic and histologic changes. Recently, a phase I clinical trial showed the initial safety profile and effect of chimeric antigen receptor (CAR)-glypican-3 (GPC3) T-cell therapy for patients with advanced HCC (58). Those CAR T cells include a humanized anti-GPC3 single-chain variable fragment, CD8α hinge domain, CD8α transmembrane domain, CD28 intracellular domain, and CD3ζ intracellular signaling domain. There are some recruiting clinical trials for investigating GPC3-targeted CAR-T Cell for treating HCC, such as trials NCT03198546 and NCT04121273.

### Gut Microbiota-Mediated Therapy

Gut microbiota has been shown to play vital roles in human liver diseases (59), through modulating secondary bile acids (BAs), activating TLRs, and influencing the function of immune checkpoint inhibitors (ICIs). For example, gut microbial extracts from NAFLD-HCC patients dramatically suppressed CD8^+^ T cells and B cells in PBMCs from non-NAFLD healthy people compared to bacterial extract from non-NAFLD controls, but significantly increased the proliferation of CD3^+^CD4^+^CD25^+^Foxp3^+^ Tregs, inducing an immunosuppressive phenomenon (20). Fecal microbiota transplantation (FMT) from proper donors can restore gut microbiota disorder and ameliorate D-galactosamine-induced liver injury in BALB/c mice, via downregulating the expression of IL-17a, TNF-α, and transforming growth factor-β (TGF-β) and upregulating the expression of IL-10 and IL-22 (60).

### miRNA-Mediated Treatment

Overexpression of microRNA-195 (miR-195) can improve the balance of Th17/Treg via regulating CD40 expression in rat liver tissues, accompanying decrease of serum level of proinflammatory cytokines (e.g., TNF-α), total cholesterol (TC) and triglyceride (TG), liver injury markers aspartate transaminase (AST), and alanine aminotransferase (ALT) (61). In addition, hepatocyte-specific overexpression of miR-34a promoted high cholesterol and fructose (HFCF) fat diet-induced NAFLD in mice, while pharmaceutical suppression of miR-34a can reverse NAFLD progression (62). miR-26a can inhibit hepatic expression of IL-17 and IL-6, as lentiviral vector delivered miR-26a treatment significantly decreased total liver weight, liver deposition of TG, and serum ALT concentration compared lentiviral control-treated mice, accompanying decreased infiltration of γδ T cells, and granulocyte-differentiation antigen-1 (Gr-1)^+^ cells and CD11b^+^ cells (63). In addition, Escutia-Gutiérrez et al. reported that miRNAs such as miR-21a-5p, miR-34a-5p, miR-122-5p, and miR-103-3p were increased expression of in livers of MAFLD/NASH (64), the potential targets for HCC treatment.

### Chemokine or Cytokine-Mediated Treatment

Treatment with C-C chemokine receptor (CCR)2 antagonist inhibited tumor-infiltrating macrophage (TAMs)-mediated immunosuppression and increased CD8^+^ T cells in liver cancer (65). In addition, this antagonist improved the therapeutic effect of sorafenib via enhancing tumor necrosis and apoptosis. CCR5/CCL5 signaling pathway plays a critical in the development of HCC in chronic liver disease both in mice and humans (66–68), as a potential treatment target for HCC. Moreover, injection of WSX1 (IL-27 receptor α) can significantly suppress the HCC growth by suppressing PD-L1 expression on tumor cells via blocking phosphoinositide 3-kinase delta (PI3Kδ)/protein kinase B (AKT)/glycogen synthase kinase-3β (GSK3β) pathway to release the cytotoxic effect of CD8^+^ T cells (69). Combined therapy with regorafenib and anti-PD-1 increased the filtration and activation of CXCR3^+^CD8^+^ T cells via increasing CXCL10 expression in tumors, resulting in inhibition of HCC growth (70). Therefore, modulating chemokines, chemokine receptors, and cytokines can improve anti-tumor immunity to inhibit tumor progression.

## Discussion

Obesity and NAFLD are closely linked with each other. NAFLD patients with medium-high risk obesity with body mass index (BMI) >35 kg/m^2^ showed poor response to hepatitis B virus (HBV) vaccine (71). In addition, hepatitis B surface antigen-specific CD4^+^ T cells showed significantly less proliferation in PBMCs of high-risk obesity NAFLD patients compared to that in low-risk obesity NAFLD subjects. Fatty liver disease also is a serious issue for obese children. Lipid metabolism is one of the major contributing factors for NAFLD (72). Fat metabolism modulates T cell profiles in the liver of NAFLD subjects to impact NAFLD-HCC progression. New technologies (e.g., siRNA-seq) improve our understanding of the pathogenesis of NAFLD. Each subtype of T cells is shown to play different roles in NAFLD progression, such as TCRαβ^+^CD3^+^CD4^−^CD8^−^ cells and CXCR6^+^CD8^+^ or PD1^+^CXCR6^+^CD8^+^ T cells. Targeting those T cells by orchestrating gut microbiota, treatment of miRNAs, adoptive transfer of T cells, and modulating the expression of small molecules are potential treatment options against NAFLD and NAFLD-HCC progression. In addition, energy restriction is a method to reduce BMI and ameliorate fatty liver disease, which may bring new health concerns. Supplement of lycopene-rich tomato juice to obese children can improve calorie-restricted regimen-induced impairment of glycolysis and mitochondrial metabolism in T cells to enhance their immune surveillance function (73).

T cell populations vary during the development of NAFLD-related HCC, including the changes in subtype and function. For example, Tregs in the early stage of NAFLD/NASH can suppress liver inflammatory function, but in the HCC stage, they can inhibit effector T cell function to suppress tumor progression. Therefore, manipulation of T cell function or population is dependent on the stage of liver disease and microenvironment. In addition, proteomic analysis of NAFLD-HCC infiltrating T cells is awaited to explore the functional proteins to modify those T cell functions except PD-1 and CXCR6. Overall, T cells play a critical role in metabolic fatty liver diseases to HCC progression, and targeting them may provide a novel treatment.

## Author Contributions

CZ and MY conceived the opinion and wrote the manuscript. All authors contributed to the article and approved the submitted version.

## Conflict of Interest

The authors declare that the research was conducted in the absence of any commercial or financial relationships that could be construed as a potential conflict of interest.

## Publisher's Note

All claims expressed in this article are solely those of the authors and do not necessarily represent those of their affiliated organizations, or those of the publisher, the editors and the reviewers. Any product that may be evaluated in this article, or claim that may be made by its manufacturer, is not guaranteed or endorsed by the publisher.
